# Diagnosis of benign and malignant thyroid nodules by a dual-layer spectral detector CT-based nomogram

**DOI:** 10.3389/fonc.2023.1132817

**Published:** 2023-03-17

**Authors:** Rongqi Yi, Ting Li, Gang Xie, Kang Li

**Affiliations:** ^1^ Department of Radiology, Chongqing General Hospital, Chongqing, China; ^2^ Department of Medical Imaging, North Sichuan Medical College, Nanchong, Sichuan, China

**Keywords:** dual-layer spectral-detector CT, thyroid nodules, nomogram, diagnosis, differentiation

## Abstract

**Introduction:**

Preoperative diagnosis of benign and malignant thyroid nodules is crucial for appropriate clinical treatment and individual patient management. In this study, a double-layer spectral detector computed tomography (DLCT)-based nomogram for the preoperative classification of benign and malignant thyroid nodules was developed and tested.

**Methods:**

A total of 405 patients with pathological findings of thyroid nodules who underwent DLCT preoperatively were retrospectively recruited. They were randomized into a training cohort (n=283) and a test cohort (n=122). Information on clinical features, qualitative imaging features and quantitative DLCT parameters was collected. Univariate and multifactorial logistic regression analyses were used to screen independent predictors of benign and malignant nodules. A nomogram model based on the independent predictors was developed to make individualized predictions of benign and malignant thyroid nodules. Model performance was evaluated by calculating the area under the receiver operating characteristic curve (AUC), calibration curve and decision curve analysis(DCA).

**Results:**

Standardized iodine concentration in the arterial phase, the slope of the spectral hounsfield unit(HU) curves in the arterial phase, and cystic degeneration were identified as independent predictors of benign and malignant thyroid nodules. After combining these three metrics, the proposed nomogram was diagnostically effective, with AUC values of 0.880 for the training cohort and 0.884 for the test cohort. The nomogram showed a better fit (all p > 0.05 by Hosmer−Lemeshow test) and provided a greater net benefit than the simple standard strategy within a large range of threshold probabilities in both cohorts.

**Discussion:**

The DLCT-based nomogram has great potential for the preoperative prediction of benign and malignant thyroid nodules. This nomogram can be used as a simple, noninvasive, and effective tool for the individualized risk assessment of benign and malignant thyroid nodules, helping clinicians make appropriate treatment decisions.

## Introduction

In recent years, with increased health awareness and the widespread use of imaging, the incidence of thyroid nodules has increased significantly, with a detection rate of approximately 65% in the general population. Although only 5%-15% of nodules are malignant (1) and mortality from thyroid cancer is stable, invasive surgical operations on thyroid nodules have increased significantly (2, 3). More importantly, there is a significant difference between clinical interventions for benign and malignant thyroid nodules (4). Therefore, to avoid overdiagnosis of thyroid nodules and overtreatment of low-risk benign nodules, preoperative noninvasive identification of benign and malignant thyroid nodules is important to plan further therapeutic approaches and define the scope of surgical intervention.

Fine-needle aspiration (FNA) cytology biopsy is the main method for the qualitative diagnosis of thyroid nodules, but it is invasive, has poor patient compliance, and has a relatively high rate of both nondiagnostic and false-negative results (5). The ultrasound (US) imaging is currently the most commonly used noninvasive test for the evaluation of thyroid nodules, and ultrasound-guided biopsy is the standard procedure for establishing the diagnosis of benign and malignant thyroid nodules noninvasively (6). However, US is dependent on operator technique and does not clearly show mediastinal lesions. Better diagnostic performance can be obtained by some functional sequences of magnetic resonance imaging (MRI), but MRI is costly, time-consuming and rarely used in clinics (7). Computed tomography (CT) can also be used to identify thyroid nodules, provide objective images, establish preoperative localization, provide information for diagnosis, and evaluate treatment outcomes. In addition, CT overcomes the drawbacks of US and FNA, including operator dependence and invasive procedures (8). Therefore, CT examinations are increasingly used for preoperative thyroid nodule evaluation.

Conventional CT is performed by a single energy technique at a certain level of tube voltage, which produces multi-color images. As a result, conventional CT relies mainly on morphological diagnosis of the lesion with few quantitative parameters and limited diagnostic efficiency (9). Dual-energy computed tomography (DECT) is an emerging CT technique that can provide quantitative measurements of substance concentration and decomposition, as well as the ability to detect specific substances (iodine, water, etc) (10). Different technological approaches can produce attenuation differences between two different photon energies, such as dual-layer detector spectral CT, the DLCT system uses two predefined detector layers to decompose a single X-ray beam into separate high- and low-energy components that are detected simultaneously. This eliminates the need to change scanning parameters (11). When DLCT is used, various quantitative parameters, such as iodine concentration (IC),the slope of spectral HU curves (λ_HU_) and effective atomic number (Zeff), can be obtained from reconstructed image sets, including virtual monoenergetic images (VMI), effective atomic number maps and material decomposition images (iodine, water, etc). Furthermore, lesions can be analyzed with the help of three-dimensional regions of interest to effectively elucidate the internal spatial heterogeneity of lesions and improve the sensitivity of their diagnosis (12). In addition, DLCT has been shown to increase the confidence of radiologists and reduce the need for additional examinations in patients with suspected malignancy (13).

Similarly, DECT has improved the noninvasive evaluation of thyroid nodules, and an increasing number of studies have indicated that quantitative parameters derived from DECT, especially quantitative IC, can distinguish benign from malignant thyroid nodules (6, 9, 14–16). However, there are few relevant studies exploring the applications of DECT, and some of them show contradictory results. Therefore, studies with larger sample sizes are needed to determine the diagnostic value of DECT for benign and malignant thyroid nodules. The aim of this study was to evaluate the potential benefit of quantitative DLCT analysis in identifying benign and malignant thyroid nodules, to develop a preoperative prediction model, and to construct a nomogram to provide clinicians with a reference for developing personalized and precise treatments.

## Information and methods

### Patient population

**Figure 1:**
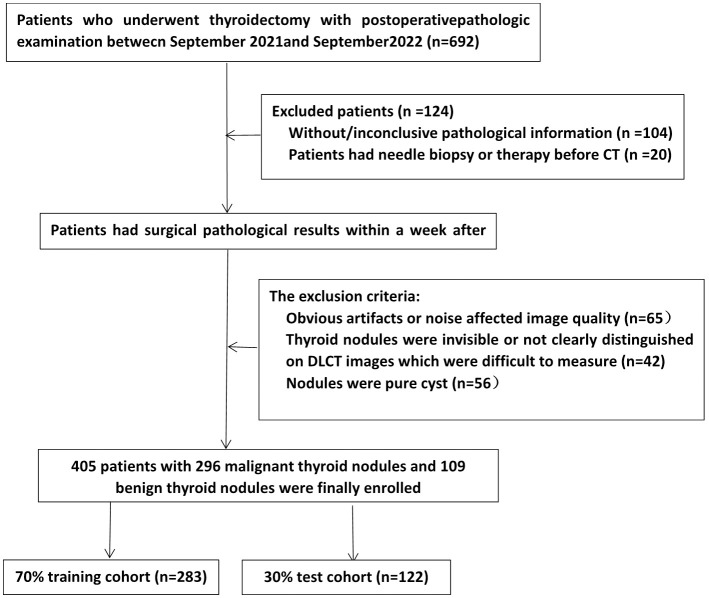
Flowchart showing the screening strategy for patients in this study.

Patients who underwent DLCT scans of thyroid nodules at our hospital between September 2021 and September 2022 were eligible for enrollment. The inclusion criteria were as follows: (1) pathological surgical findings within one week of the CT examination were available; (2) nodules were solid or mixed cystic; and (3) when more than two nodules were present in a lobule, only the largest nodule was selected. The exclusion criteria were as follows: (1) the presence of significant CT artifacts; (2) the patient had undergone a puncture biopsy or other treatment prior to CT examination; and (3) nodules were difficult to measure or to distinguish on DLCT images. The screening flow chart is shown in **Figure 1**. Finally, a total of 405 consecutive patients were retrospectively included. We divided them into training and test groups using stratified randomization in a 7:3 ratio. Demographic information, including sex and age, from medical records was reviewed.

### Image acquisition

All patients were imaged using a DLCT scanner (IQon Spectrum CT; Philips Healthcare, The Netherlands) in the neck region. The sweep ranged from the base of the skull to the aortic arch. The parameters were as follows: tube voltage, 120 kV; tube current, smart mAs; rotation time, 0.5 s; detector collimation, 64 × 0.625 mm; field of view, 350 × 350 mm; matrix, 512 × 512; layer thickness, 5 mm; and reconstruction thickness, 1.25 mm. The scanning parameters were set according to the lowest possible level of radiation for safety. After acquisition of nonenhanced images, iodinated nonionic contrast agent (iophorol; 350 mg/mL) was used. The iodine dose was 1.5 mL/kg at a flow rate of 3.5 mL/s for a total injection volume of 60-70 mL and was followed by a single injection of 30 mL of saline at the same flow rate using a double-ended syringe through the right median elbow vein. Arterial phase (AP) scan timings were determined by an automatic trigger technique with a 40-second delay in the AP. The delay before the venous phase (VP) scan was 30 seconds after the end of the arterial scan.

The spectral data were transferred to an external workstation for further analysis. Arterial and portal vein phase acquisitions for each examination were generated using the vendor’s image postprocessing tool (InteliSpace Portal Version 10.1, Philips Healthcare, Best, The Netherlands) and a dedicated spectral reconstruction algorithm to generate VMI reconstructions (energy levels from 40 keV to 200 keV), substance breakdown maps of iodine concentration and Zeff maps.

### Image postprocessing and analysis

Independent analysis was performed by two experienced radiologists. Although they were informed that all patients had thyroid nodules, the observers were unaware of other relevant clinical data and pathological findings.

First, conventional imaging features of thyroid nodules, including nodule laterality (left lobe, right lobe, or isthmus) and location (superior, middle, or inferior), calcification (positive, negative), margins (clear, blurred), cystic degeneration (positive, negative), and diameter were obtained. Next, ROIs were placed in the largest cross-section of the nodule, taking care to avoid obvious necrosis, vascularity, calcification, and cystic areas. Attenuation values were measured for plain, AP and VP CT scans, and the enhancement rate was calculated (enhanced CT value– plain CT value)/plain CT value). In addition, the effective atomic number and IC values were measured in the arterial and venous phases. Notably, the IC generated from the iodine-based material decomposition images was normalized to the IC in the common carotid artery by first placing the ROI on the same slice. Then, the normalized IC was derived by dividing the IC in the node by the IC in the common carotid artery (NIC). The NIC was then further classified into NIC in the AP (NIC-A) and NIC in the VP (NIC-V). The formula was calculated as follows:


NIC=IC of nodule/IC of the aorta

The slope of the spectral HU curves was quantified on single-energy AP and VP images. Finally, spectral curves were obtained from 40 to 100 keV monochromatic energy images. The slope of the spectral HU curves was calculated by the following equation:


$${\text{λ}}_{\text{HU}}=\left(\text{CT value at }40\text{ keV}-\text{CT value at }100\text{ keV}\right)/\left(100-40\right)$$

The Zeff was automatically calculated by the software.

### Statistics and models

Statistical analyses were performed using SPSS (version 26.0) and R software (version 4.1.0). P values less than 0.05 were considered statistically significant. The distribution of data for each group was determined by the Kolmogorov−Smirnov test. Continuous variables that conformed to a normal distribution are expressed as the mean ± standard deviation (s); otherwise, they are expressed as the median and interquartile range [M (Q1, Q3)]. Categorical variables are expressed as the frequency (n) and composition ratio (%). For between-group analysis, the independent sample t test or Mann−Whitney U test was used for continuous variables, and the Wilcoxon rank-sum test or Fisher’s exact test was used for categorical variables. Univariate and multivariate logistic regression with a 95% confidence interval (CI) was used to estimate the odds ratio (OR) and to identify independent predictors of benign and malignant nodules. Prediction models were developed using the screened independent predictors, and validated in the test cohort, nomogram was constructed, and receiver operating characteristic (ROC) curves and AUC, sensitivity, specificity, positive predictive value (PPV), and negative predictive value (NPV) were also calculated with 95% confidence intervals. Additionally, the calibration curve was used to evaluate the model fit, and DCA was used to evaluate the net clinical benefit of the model.

## Results

### Clinical characteristics and DLCT parameters

According to the histological diagnosis, there were 296 patients with malignant thyroid nodules (281 papillary carcinomas, 8 medullary carcinomas, 6 follicular carcinomas, and 1 eosinophilic carcinoma) and 109 patients with benign thyroid nodules (60 nodular goiter, 13 nodular goiter with adenomatous hyperplasia, 19 granulomatous thyroiditis, 14 follicular adenomas, 1 fibrotic nodule, 1 focal lymphoid hyperplasia, and 1 lymphatic follicular inflammation) in the study population. The training cohort consisted of 283 patients (42 males and 241 females, mean age 42.40 years ± 11.47), and the test cohort consisted of 122 patients (24 males and 98 females, mean age 43.04 years ± 12.50). The demographic data and DLCT parameters for the training and test cohorts are shown in **Table 1**.

**Table 1:** Clinical Characteristics and DLCT Parameters of Patients in Training and Test Cohorts.

	Training cohort (n=283)	Test cohort (n=122)	P value
Age, y	42.40 ± 11.47	43.04 ± 12.50	0.326
Sex (male)	42 (14.84%)	24 (19.67%)	0.289
Nodule position			0.306
Inferior	61 (21.56%)	31 (25.41%)	
middle	152 (81.64%)	69 (56.56%)	
Superior	70 (24.73%)	22 (18.03%)	
Nodule laterality			0.783
Left lobe	155 (54.77%)	67 (54.91%)	
Right lobe	112 (43.11%)	46 (45.90%)	
isthmus	16 (0.565%)	9 (0.73%)	
Size (cm)	1.14 ± 0.56	1.06 ± 0.51	0.523
Margin (Unclear)	109 (38.52%)	51 (41.80%)	0.610
Calcification	78 (27.56%)	38 (31.15%)	0.662
Cystic degeneration	30 (10.60%)	8 (6.56%)	0.274
Enhanced blurring	95 (33.57%)	39 (31.97%)	0.842
Enhancement rate-A	1.31 ± 1.11	1.40 ± 1.45	0.479
Enhancement rate-V	1.26 ± 0.84	1.29 ± 1.09	0.486
IC-A	2.73 ± 1.43	2.76 ± 1.53	0.429
NIC-A	0.28 ± 0.14	0.28 ± 0.13	0.464
Zeff-A	8.74 ± 0.59	8.74 ± 0.59	0.416
λHu-A	3.65 ± 1.73	3.69 ± 1.84	0.448
IC-V	2.65 ± 1.06	2.56 ± 0.84	0.428
NIC-V	0.66 ± 0.29	0.64 ± 0.20	0.323
Zeff-V	8.69 ± 0.51	8.67 ± 0.36	0.362
λHu-V	3.51 ± 1.30	3.37 ± 1.06	0.329

IC-A, Iodine concentration in the arterial phase; NIC-A, Standardized iodine concentration in the arterial phase; λHu-A, Slope of the spectral Hounsfield unit curve in the arterial phase; Zeff-A, Effective atomic number in the arterial phase; IC-V, Iodine concentration in the venous phase; NIC-V, Standardized iodine concentration in the venous phase; λHu-V, Slope of the spectral Hounsfield unit curve in the venous phase; Zeff-V, Effective atomic number in the venous phase.

### DLCT-based nomogram development and validation

**Figure 2:**
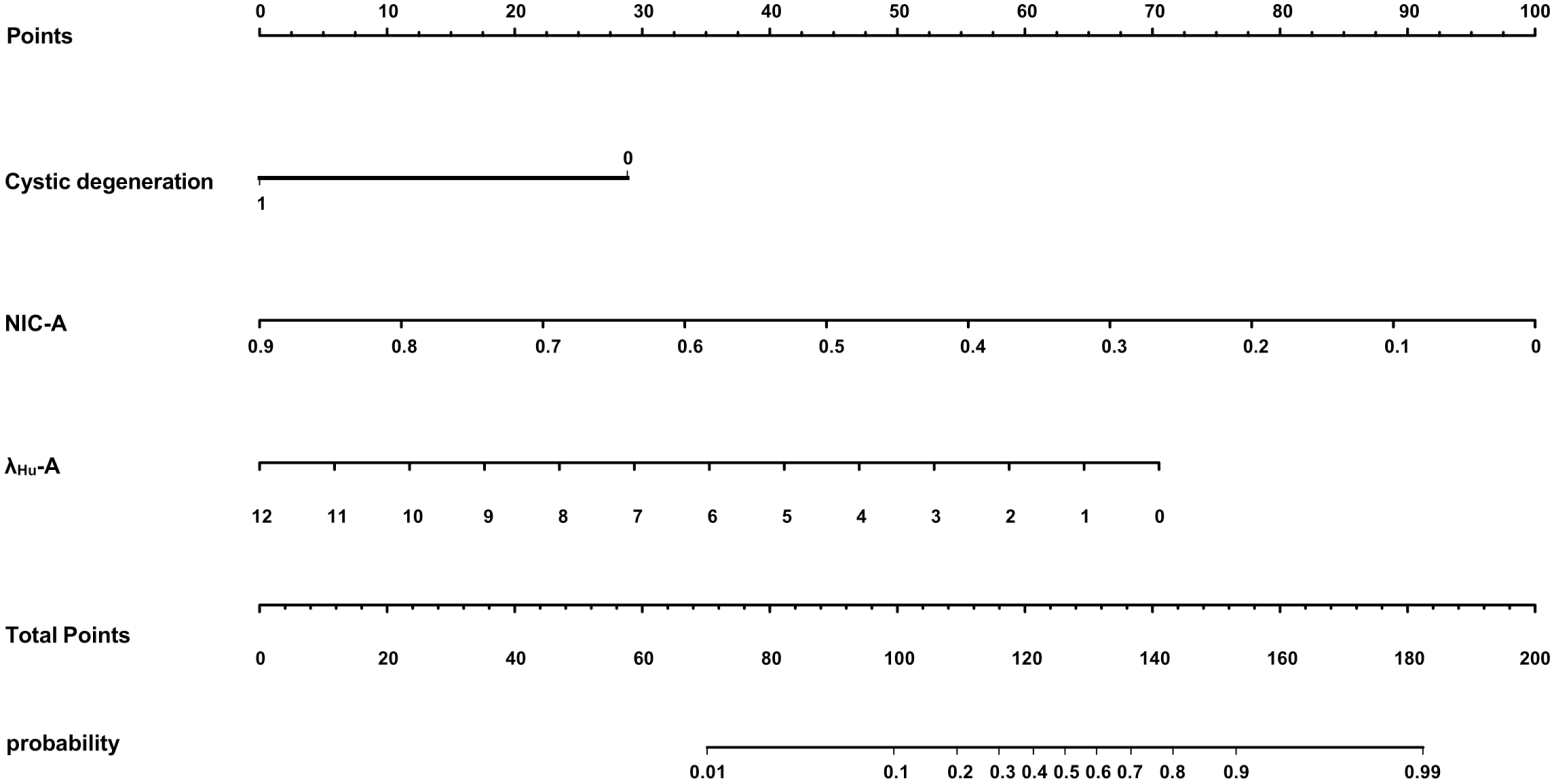
A DLCT-based nomogram was plotted combining independent radiological features with DLCT quantitative parameters in the training cohort.

Variables with p<0.05 in the univariate analysis were included in the multivariate logistic regression analysis. The data indicated that cystic degeneration, NIC-A and λ_HU_-A were significant predictors of benign and malignant thyroid nodules after correction for common factors (**Table 2**). Subsequently, a DLCT-based nomogram was developed using the three final factors and their derived regression coefficients to predict the benignity and malignancy of the thyroid nodules (**Figure 2**).

**Table 2:** Univariate Logistic Regression and Multivariate Logistic Regression Results for Training Cohorts.

	Univariate analysis		Multi variate analysis	
	OR (95%CI)	P value	OR (95%CI)	P value
Age, y	1.007 (0.98-1.03)	0.552	–	–
Sex(male)	1.207 (0.56-2.59)	0.630	–	–
Nodule position	1.023 (0.70-1.51)	0.908	–	–
Nodule laterality	0.937 (0.60-1.48)	0.778	–	–
Size(cm)	1.471 (0.94-2.23)	0.090	–	–
Margin(Unclear)	0.516 (0.29-0.92)	0.024	–	–
Calcification	0.682 (0.37-1.25)	0.217	–	–
Cystic degeneration	10.134 (4.27-24.0)	0.000	10.593(3.31-33.95)	0.000
Enhanced blurring	0.477 (0.26-0.88)	0.017	–	–
Enhancement rate-A	2.795 (1.94-4.03)	0.000	–	–
Enhancement rate-V	2.031 (1.44-2.86)	0.000	–	–
IC-A	3.139 (2.31-4.27)	0.000	–	–
NIC-A	3.723 (2.65-5.23)	0.000	2.484(1.57-3.93)	0.000
Zeff-A	10.139 (5.18-19.9)	0.000	–	–
λHu-A	2.650 (2.05-3.43)	0.000	1.618(1.10-2.39)	0.016
IC-V	2.068 (1.57-2.72)	0.000	–	–
NIC-V	19.505 (6.03-63.07)	0.000	–	–
Zeff-V	4.410 (2.29-8.51)	0.000	–	–
λHu-V	1.611 (1.30-1.99)	0.000	–	–

IC-A, Iodine concentration in the arterial phase; NIC-A, Standardized iodine concentration in the arterial phase; λHu-A, Slope of the spectral Hounsfield unit curve in the arterial phase; Zeff-A, Effective atomic number in the arterial phase; IC-V, Iodine concentration in the venous phase; NIC-V, Standardized iodine concentration in the venous phase; λHu-V, Slope of the spectral Hounsfield unit curve in the venous phase; Zeff-V, Effective atomic number in the venous phase.

**Figure 3:**
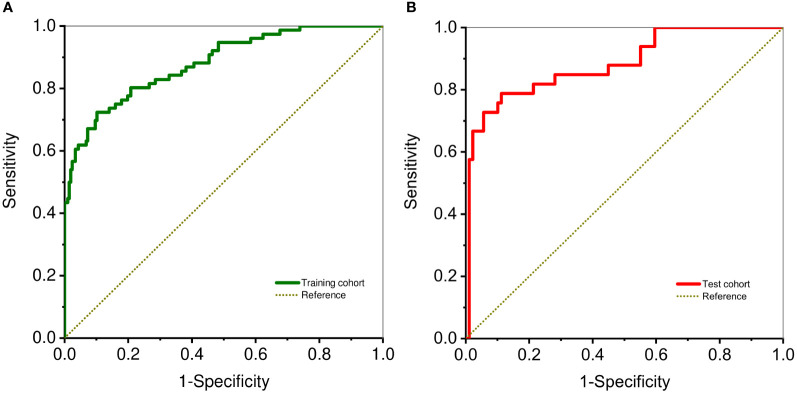
ROC curve analysis shows diagnostic ability DLCT-based nomogram in training **(A)** and test cohorts **(B)**.

**Figure 4:**
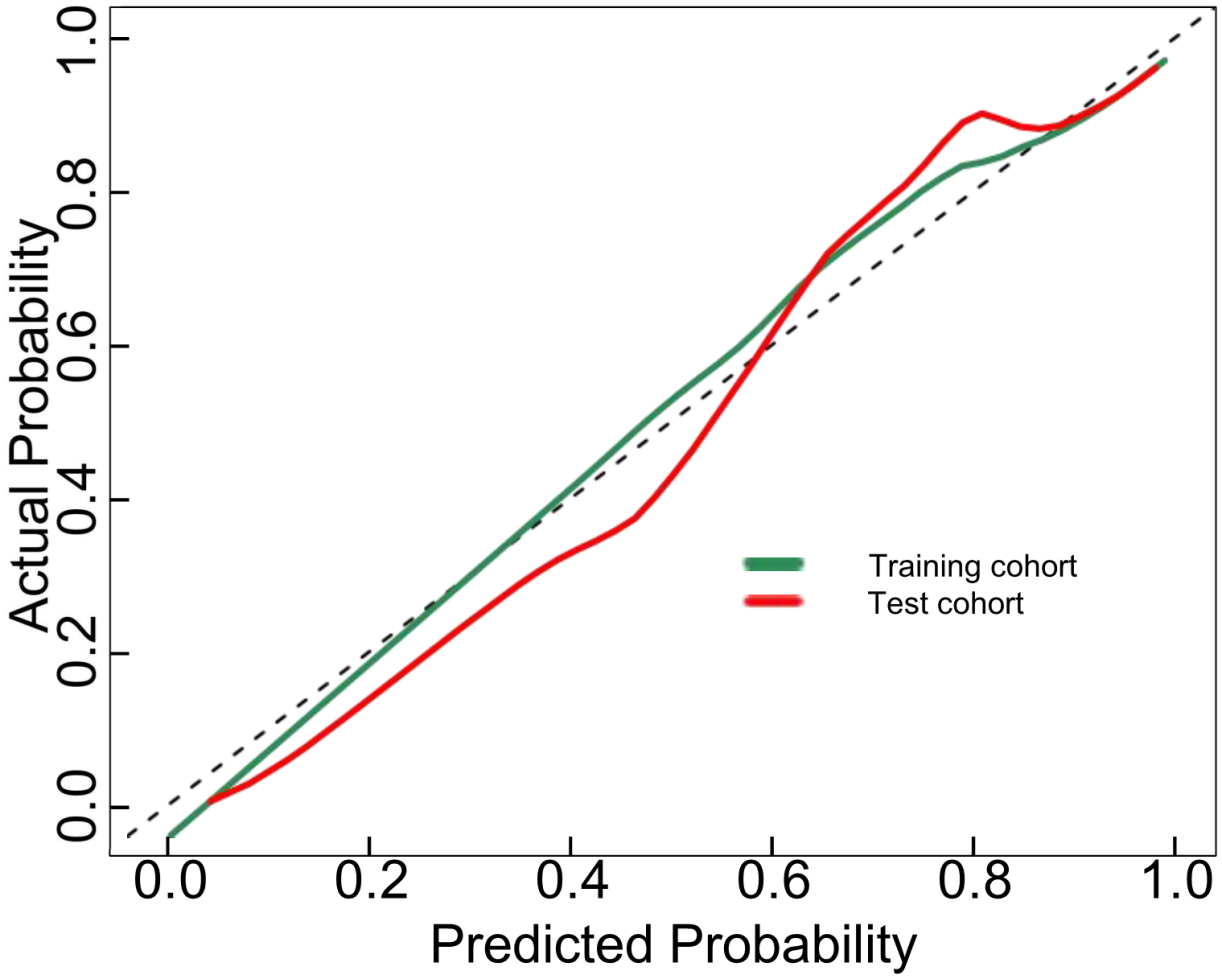
Calibration curves for the DLCT-based nomogram in the training and test cohort. The 45°straight line indicates the ideal performance of the DLCT-based nomogram. A closer distance between two curves indicates higher accuracy.

The proposed nomogram showed good diagnostic power with an AUC of 0.880 (95% CI 0.834-0.927) for the training cohort. Meanwhile, we used the bootstrap method to internally validate the model, after 200 iterations, the model’s average AUC of 0.881 (95% CI 0.877-0.883) in the training cohort, indicating that the model coefficients were stable. And finally, the AUC in the test cohort was 0.884(95% CI 0.811-0.956). The ROCs of the prediction are shown in **Figure 3**. In the training cohort, the calculated sensitivity and specificity were 0.95 and 0.62, with a PPV and NPV of 0.87 and 0.82, respectively, while in the test cohort, the sensitivity and specificity were 0.97 and 0.67, with a PPV and NPV of 0.89 and 0.88, respectively (**Table 3**). In addition,the calibration curves showed good agreement between the nomogram-predicted and actual probabilities in both cohorts (**Figure 4**).

**Table 3:** The DLCT-based Nomogram Performance Results.

	Training cohort (n=283)	Test cohort (n=122)
Sensitivity	0.95	0.97
Specificity	0.62	0.67
Positive predictive value (PPV)	0.87	0.89
Negative predictive value(NPV)	0.82	0.88
Area under the curve(AUC)	0.880	0.884

**Figure 5:**
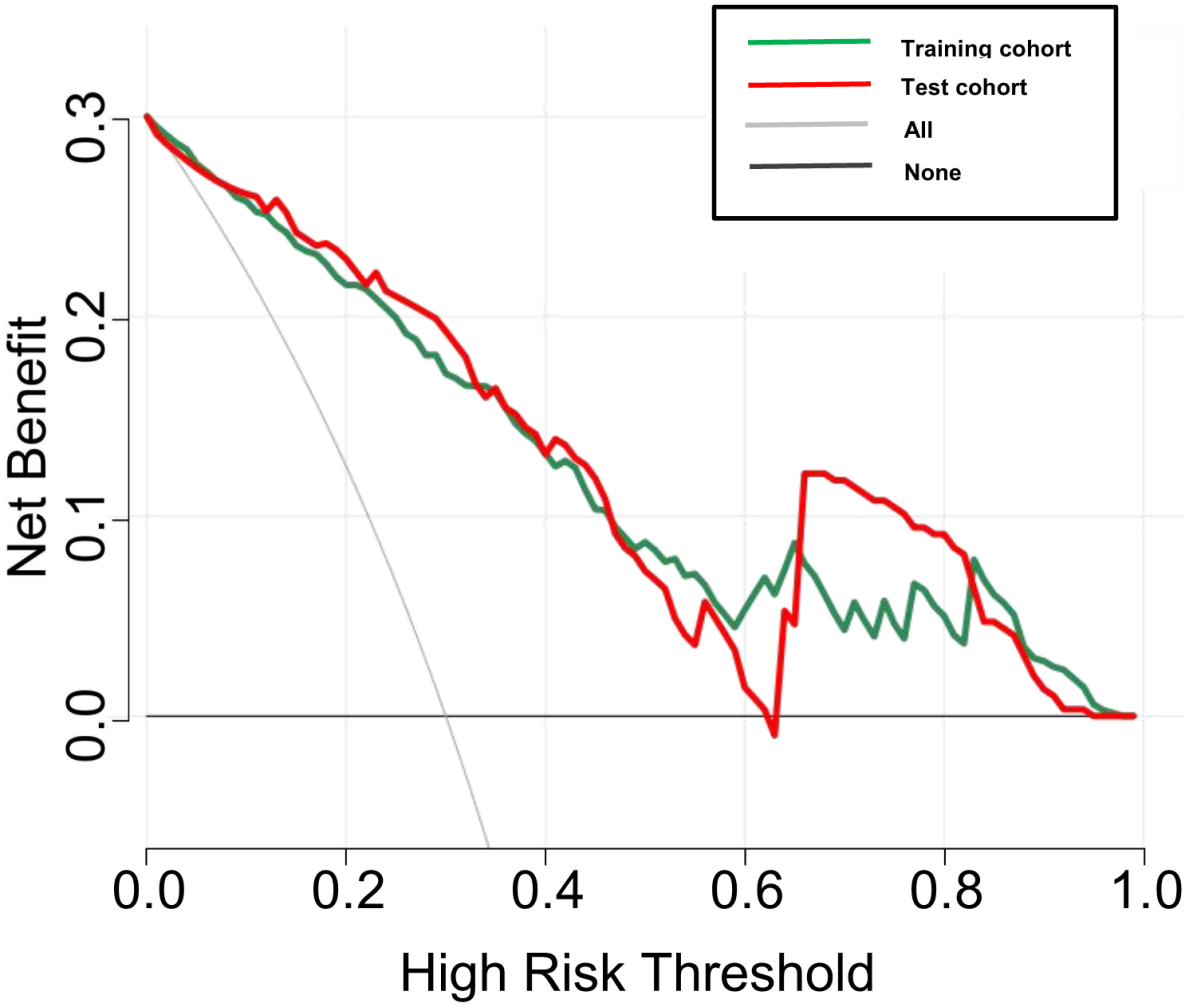
Decision curve analysis (DCA) for the DLCT-based nomogram in the training and test cohort. The y-axis measures the net benefit and the x-axis represents the threshold probability.

**Figure 6:**
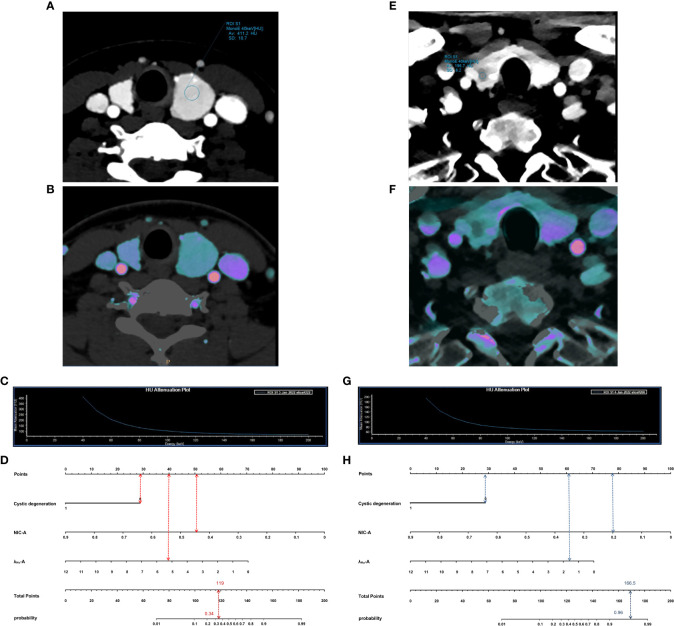
Examples of application of nomogram to predict probability of benign and malignant thyroid nodules. Each nomogram shows value of each predictor on axis for variable and its corresponding score on points scale. When points for all variables were added, total scores and corresponding risk probability were superimposed on scales showing total points and probability (risk). **(A–D)**, 54-year-old man with a benign thyroid nodule. Arterial phase 40-keV monochromatic image **(A)**, Iodine density image **(B)** and the slope of the spectral HU curve **(C)** show lesions. When points for individual predictors were added, total points were 119. Nomogram **(D)** shows that the probability of malignancy of this lesion was 0.34. E–H, 36-year-old woman with a malignancy thyroid nodule. Arterial phase 40-keV monochromatic image **(E)**, Iodine density image **(F)** and the slope of the spectral HU curve **(G)** show lesions. When points for individual predictors were added, total points were 166.5. Nomogram **(H)** shows that the probability of malignancy of this lesion was 0.96.

DCA was used to examine the benefits of the developed nomogram for predicting benign and malignant thyroid nodules. The results showed that our model had a net benefit between 0 and 1. We also found that the nomogram had a net benefit for most threshold probability ranges in the test cohort (**Figure 5**). **Figure 6** shows two examples of the nomogram predicting benign and malignant thyroid nodules.

## Discussion

In this study, we used quantitative DLCT analysis to preoperatively predict whether thyroid nodules were benign or malignant. Previous studies have shown that DLCT parameters are highly correlated with the nature of thyroid nodules. To better understand the value of these features for the preoperative prediction of benign and malignant thyroid nodules, we built and tested a DLCT-based nomogram. Our study shows that the proposed nomogram is a promising tool for preoperative prediction of thyroid nodule nature, rapidly providing strong evidence for targeted treatment and personalized management of thyroid nodule patients.

Our data show that malignant thyroid nodules were much less likely to develop cystic degeneration, which is largely consistent with the findings of previous studies (17, 18) showing that more than 90% of thyroid cancers lack cystic degeneration, while nodular goiters often show cystic degeneration due to the relative abundance of glial follicles and hemorrhage. Nodular goiters were the predominant pathological type of benign thyroid nodules in this study. These observations suggest that malignant thyroid nodules are less likely to develop cystic degeneration than benign nodules. Importantly, in the multivariate analysis, cystic degeneration was identified as an independent differentiator of benign and malignant thyroid nodules and was therefore added to the nomogram. In addition, some studies (1, 9) have showed that calcification and enhancement blurring are more common in malignant nodules than benign nodules. However, these findings were not corroborated in the present study. Considering the conflicting observations, a prospective study with a larger cohort is needed to obtain conclusive results.

Previous studies (19) reported that DLCT quantitative parameters, especially IC values, can be used to diagnose malignant and benign tumors. In conventional contrast-enhanced CT, the mean CT value (in units of HU) is the result of a combination of the degree of iodine enhancement and the CT value of the underlying tissue. Therefore, quantification of IC is difficult in areas with mixed tissue types. However, in DLCT, IC can be measured based on iodine uptake per unit volume by iodine-based material decomposition imaging, which is a more realistic representation of IC values (20, 21).Tang et al. (22) demonstrated through simulation experiments that quantitative IC values derived from DECT are highly consistent with actual iodine deposition in the tissue, suggesting that they represent the true distribution of iodine in the tumor tissue. Following efforts to minimize individual circulation-induced variability, NIC has been shown to be an important marker in a wide range of clinical cancer studies (22–25). Dohán et al. (26) reported a decrease in iodine intake in thyroid cancer. This finding was confirmed by a series of reports and subsequently used to differentiate malignant from benign thyroid nodules. Lee et al. (16) reported that 76 patients with thyroid nodules had lower (p< 0.05) DECT-based standardized IC values for malignant thyroid nodules in the arterial phase compared to benign nodules, indicating that spectral CT could be used to assess abnormal iodine levels in patients with thyroid nodules. Similarly, in a retrospective study that included 150 patients with thyroid nodules, benign nodules examined with spectral CT exhibited higher iodine concentrations and standardized IC in the arterial phase, suggesting that IC values from spectral CT are a useful biomarker for differentiating malignant from benign nodules (8).

This is generally consistent with our finding that NIC-A was both lower in malignant thyroid nodules and statistically significant in a multifactorial analysis (p< 0.001). The DLCT parameters of thyroid nodules reflect the sum of nodal microvascular density and blood supply as well as intrinsic iodine uptake by thyroid tissue (27). Previous studies have confirmed that among the many factors affecting thyroid iodine content, the most relevant are intrinsic thyroid-related factors, including follicular cell counts and iodine transport proteins in the cell membrane (16). In malignant nodules, follicular cells are replaced by cancer cells or fibrous tissue, and iodine uptake capacity is significantly reduced or even absent, resulting in lower iodine uptake rates. In contrast, nodular goiters and adenomas have normal follicular structure and iodine uptake capacity. For this reason, the iodine density values in the malignant group were significantly lower than those in the benign group. Recently, Jiang et al. (14) demonstrated that the IC of malignant nodules was significantly lower in nonenhanced CT images and higher in contrast-enhanced CT images than that of benign nodules. They hypothesized that the IC of thyroid tissue reflects iodine uptake function on nonenhanced CT images and blood supply on enhanced CT images; however, this is different from our findings. One possible explanation is the difference in the included samples, when the increased IC caused by the blood supply of malignant nodules may partially offset the decreased iodine uptake capacity of thyroid cancer, resulting in the increased IC. In addition, measurement factors and DLCT scan parameters are nonnegligible causes of potential error.

Virtual monochromatic imaging allows the characterization of tissues by studying their attenuation of X-rays at continuous energy levels. The dependence of attenuation on photon energy then means that the spectral curves of different tissues can be analyzed to quantitatively distinguish between different tissue types. One study reported that the slope of the spectral HU curves have similar sensitivity (91.1% vs. 83.0%) in assessing cancer as conventional CT methods (28). Our results showed that λ_HU_-A was lower in the malignant group than in the benign group, which is consistent with previous findings (16), and is possibly driven by the presence of lipid material in thyroid cancer cells (29), which may be characteristic of the malignant phenotype.

According to these previous reports, it is reasonable to expect lower values of NIC-A and λ_HU_-A in malignant nodules than in benign nodules; NIC-A and λ_HU_-A may be two valid indicators for assessing the benignity of thyroid nodules. Therefore, in addition to qualitative imaging features (cystic degeneration), quantitative DLCT parameters (NIC-A and λ_HU_-A) were included in the predictive nomogram model. Notably, the derived nomogram allowed us to distinguish benign and malignant thyroid nodules with good performance. In addition, we performed a decision curve analysis and found a clear net benefit of the nomogram for basic clinical decision making. To our knowledge, this is the first preoperative prediction of benign and malignant thyroid nodules using a DLCT-based nomogram. These DLCT-based imaging markers are associated with more appropriate treatment modalities and clinical management of patients with thyroid nodules.

Our study has some limitations. First, this study is a single-center design, and a suitable external validation cohort is needed to further validate our results. In addition, although the National Comprehensive Cancer Network (NCCN) 2018 edition guidelines clearly state (30) that the use of iodine contrast does not cause harm to patients with thyroid cancer, it may lead to a potential delay in administration of postoperative radioactive iodine (RAI) therapy. Therefore, the timing of radioactive iodine therapy in patients after surgery needs to be appropriately adjusted. Third, radiation exposure during dual-phase contrast-enhanced DLCT scans should not be ignored. Future effective techniques are needed to reduce the radiation dose. In addition, functional indicators such as TSH serum levels (31), which have been shown to be associated with malignant thyroid nodules, were not included in this study because these measures were not available in a small subset of targeted patients. Finally, we used only pure image data, and better prediction results may be achieved if radiomics or machine learning can be combined with the nomogram.

## Conclusion

In conclusion, we proposed a DLCT-based nomogram that demonstrated good predictive power for the benign and malignant thyroid nodules. The easily accessible nomogram showed excellent discriminatory and calibration capabilities in identifying benign and malignant thyroid nodules by using three noninvasively obtained variables. Thus, the predictive model can help clinicians quickly and confidently determine a plan for further clinical management and avoid unnecessary invasive procedures. Nomograms can be used as an easy to use, noninvasive and practical quantitative tool for the identification of benign and malignant thyroid nodules. In short, the nomogram holds great promise in helping to identify risky thyroid nodules.

## Data availability statement

The raw data supporting the conclusions of this article will be made available by the authors, without undue reservation.

## Ethics statement

The studies involving human participants were reviewed and approved by the Ethics Committee of Chongqing People’s Hospital. Written informed consent for participation was not required for this study in accordance with the national legislation and the institutional requirements.

## Author contributions

Study concept and design: RY and KL. Data collection: RY and TL. Data analysis and interpretation: GX and RY. and manuscript drafting and critical revisions: RY, TL and GX. All authors contributed to the article and approved the submitted version.
